# A multimodal approach to diagnosis of neuromuscular neosporosis in dogs

**DOI:** 10.1111/jvim.17145

**Published:** 2024-07-17

**Authors:** Vanessa Alf, Federica Tirrito, Andrea Fischer, Rodolfo Cappello, Anna‐Mariam Kiviranta, Tanja A. Steinberg, Federica Poli, Felix Stotz, Omar V. Del Vecchio, Stefanie Dörfelt, Cristian Falzone, André Knittel, Shenja Loderstedt, Edy Mercuriali, Joana Tabanez, Paolo Zagarella, Kaspar Matiasek, Marco Rosati

**Affiliations:** ^1^ Section of Clinical and Comparative Neuropathology Ludwig‐Maximilians‐Universität Munich Germany; ^2^ Clinica Neurologica Veterinaria NVA Milano Italy; ^3^ AniCura Istituto Veterinario di Novara Novara Italy; ^4^ Section of Neurology, Centre for Clinical Veterinary Medicine Ludwig‐Maximilians University Munich Germany; ^5^ North Downs Specialist Referrals Bletchingley UK; ^6^ Department of Equine and Small Animal Medicine, Faculty of Veterinary Medicine University of Helsinki Helsinki Finland; ^7^ Neurology Referral Service AniCura Tierklinik Haar Haar Germany; ^8^ Clinica Veterinaria Valdinievole Monsummano Terme Italy; ^9^ EVIDENSIA Tierarztpraxen und ‐kliniken Nordrhein GmbH Düsseldorf Germany; ^10^ Centro Veterinario Caleidos Albisola Superiore Italy; ^11^ Clinica Veterinaria Pedrani Diagnostica Piccoli Animali Zugliano Italy; ^12^ Klinik für Kleintiere – Chirurgie Universität Gießen Gießen Germany; ^13^ Neurologie – Klinik für Kleintiere Universität Leipzig Leipzig Germany; ^14^ Instituto Veterinario di Novara Novara Italy; ^15^ Fitzpatrick Referrals Surrey UK; ^16^ Centro Traumatologico Ortopedico Veterinario Arenzano Italy

**Keywords:** histopathology, immunohistochemistry, in situ hybridization, muscle biopsy, *Neospora caninum*, PCR

## Abstract

**Background:**

Early diagnosis of neosporosis in dogs is challenging.

**Objectives:**

To evaluate the feasibility of a compound multimodal testing approach for diagnosing in dogs neuromuscular and combined forms of neosporosis.

**Animals:**

A total of 16 dogs diagnosed with solely neuromuscular neosporosis or with a combination of neuromuscular and central nervous system neosporosis.

**Methods:**

Retrospective review of clinical signs, laboratory findings, treatment, and outcome with focus on the diagnostic utility of different tests. Development of a chromogenic in situ hybridization (ISH) assay for the identification of *Neospora caninum* in paraffin‐embedded muscle samples.

**Results:**

13/16 dogs had only neuromuscular signs of neosporosis, 3/16 had disease signs with concomitant central nervous system (CNS) involvement. Serology was performed in 15/16, with 10/15 showing titers >1 : 160 at admission. PCR on muscle samples detected *N. caninum* DNA in 11/16. Immunohistochemistry (IHC) detected *N. caninum* in 9/16 and ISH in 9/16. Histopathology revealed inflammatory myopathy in 10/16, necrotizing myopathy in 5/16, borderline changes in 1/16 and tachyzoites in 9/16. In 4 cases, *N. caninum* infection was confirmed with all 5 diagnostic methods, 3 cases with 4, 2 with 3, 6 with 2, and 1 animal with 1.

**Conclusions and Clinical Importance:**

Diagnosis of *N. caninum* infection should rely on a multimodal diagnostic approach and negativity of 1 single test should not allow for exclusion. Serology in combination with direct parasite identification via histopathology, DNA via PCR, or both modalities, appears a reliable diagnostic approach.

**Abbreviations**
ALT: alanine aminotransferase
AST: aspartate aminotransferase
BLAST: Basic Local Alignment Search Tool
CK: creatine kinase
CNS: central nervous system
CSF: cerebrospinal fluid
FFPE: formalin‐fixed paraffin‐embedded
HE: hematoxylin and eosin
IFAT: immunofluorescent antibody test
IgG: immunglobulin G
IHC: immunohistochemistry
ISH: in situ hybridization
NFT: nerve fiber teasing
PBS: phosphate‐buffered saline
PNS: peripheral nervous system
TSO: trimethoprim/sulfadizine
WBC: white blood count

## 1 INTRODUCTION

Neosporosis is a disease caused by the obligate intracellular apicomplexan parasite *Neospora caninum* that is closely related to *Toxoplasma gondii*.^1^, ^2^ The cyst‐forming apicomplexan infects a wide range of warm‐blooded animals, including livestock and companion animals.^3^ Canidae serve as intermediate, definite, or both types of hosts of *N. caninum*, and seroprevalence rates in central Europe range from 2.1% to 36.4% in dogs.^4^, ^5^, ^6^, ^7^

Usually, an infection with *N. caninum* occurs without clinical signs in adult, immunocompetent healthy dogs.^8^ The balance between invading and multiplying tachyzoites and the host's immune reaction causes cell destruction, inflammation, and development of clinical signs. Puppies and young dogs are more vulnerable resulting in severe infections and clinical presentations that are most severe when dogs are infected transplacentally.^3^, ^9^ Tachyzoites and tissue‐cysts can be found in the brain, spinal cord, nerve roots, peripheral nerves, and muscles of infected dogs. Congenitally infected puppies might develop severe lesions such as myonecrosis, encephalitis, and myocarditis, that might result in sudden death.^3^ Neosporosis infection in adult dogs causes a variety of neurological clinical signs reflecting involvement of the central nervous system (CNS), including intention tremors, paresis, ataxia, paralysis, or the neuromuscular system with paresis and rhabdomyolysis. Involvement of both, CNS and peripheral nervous system (PNS), is also described.^1^, ^8^, ^9^, ^10^, ^11^

Early detection of the disease might be difficult because of the wide range of clinical signs, exposure, and immune‐status of the dog to *N. caninum*, and its close relationship to toxoplasmosis.^2^, ^8^ Direct and indirect methods are commonly used to diagnose neosporosis. Besides a comprehensive clinical examination, clinicopathologic investigation might be suggestive of infection. Neospora‐induced myositis and myonecrosis can cause increased serum creatine kinase (CK), aspartate‐aminotransferase (AST) activities.^12^ Imaging can be helpful in locating lesions, disease extent, and sites for suitable biopsies. Detection of *Neospora* antibodies is a quick, comparatively cheap indirect detection method that can be applied antemortem.^8^

Other diagnostic modalities address the direct identification of *N. caninum* obtainable through histopathology or DNA isolation, via PCR from cerebrospinal fluid (CSF), or analysis of tissue biopsies.^13^, ^14^, ^15^

In situ hybridization (ISH) is a highly sensitive technique that localizes specific nucleotide sequences in cells and tissues using chromogenic probes.^16^, ^17^ The ISH can identify parasites,^18^, ^19^ viruses,^20^, ^21^, ^22^, ^23^, ^24^ and bacteria^25^, ^26^ in the heart,^18^, ^19^, ^20^, ^24^ smooth,^20^, ^21^, ^23^ or striated muscles.^22^, ^23^, ^26^ To the authors' knowledge, ISH has never been reported for the detection of *N. caninum* in paraffin‐embedded muscle samples.

Delayed diagnosis and treatment worsen outcome and prognosis.^27^ This study retrospectively compared the diagnostic utility of 5 test modalities including, serology, histopathology, PCR, IHC, and ISH in a cohort of dogs affected by neurologically symptomatic neosporosis. Moreover, we established oligonucleotide probes and an ISH assay for the detection of *N. caninum* with light microscopy for the evaluation of muscle biopsies.

## 2 MATERIALS AND METHODS

### 2.1 Case selection

**FIGURE 1:**
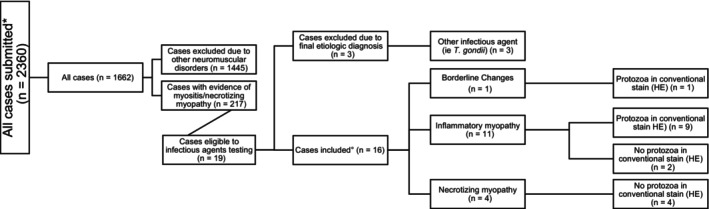
Selection process of included and excluded cases.

Cases with a diagnosis of neosporosis were selected from the archive of muscle and nerve biopsies of the Section of Clinical and Comparative Neuropathology, LMU Munich submitted between 2012 and 2022. Dogs were included based on the following criteria: (a) neurologic signs compatible with neuromuscular localization with or without concomitant CNS involvement (Table 1); (b) histopathologic diagnosis of necrotizing myopathy/myositis with or without apicomplexan parasite identification on hematoxylin and eosin (HE); (c) suspicion of *N. caninum* infection raised either by the clinician or the pathologist; (d) evidence of *N. caninum* infection with either serology (immunofluorescent antibody test [IFAT] considered positive for titers >1 : 160) or direct methods such as parasite identification on conventional HE stain, IHC, ISH, and positive PCR from submitted muscle biopsies. Dogs were excluded if they had: (a) histopathologic diagnosis of necrotizing myopathy/myositis because of other infectious or noninfectious conditions; (b) exhausted biopsy samples precluding further IHC, ISH, and PCR investigations. Signalment, history, clinical signs, laboratory findings, treatment, and outcome of the selected cases were collected and reviewed. The selection process of included and excluded cases is visualized in Figure 1. Follow‐up information was obtained by email contact.

**TABLE 1:** Summary of clinical signs in this cohort of 16 dogs diagnosed with neosporosis.

Clinical sign/finding	Number of cases
Neuromuscular	
Decreased spinal reflexes	12
Tetraparesis	9
Progressive weakness	6
Muscle loss	5
Myalgia	5
Exercise intolerance	4
Gait abnormalities	4
Nonprogressive lameness	2
Paraplegia	1
Muscle contracture	1
Rigidity of the limbs	1
Hypometria	1
Central nervous system	
Cerebellar ataxia	2
Horner syndrome	2
Loss of menace response	2
Hypermetria	2
Generalized tremor	1
Vestibular syndrome	1
Head tremor	1
Facial nerve deficits	1
Forebrain symptoms	1
Reduced swallowing reflex (puppy)	1
Other clinical findings	
Megaesophagus	3
Hyperthermia	2
Enteropathy	2
Hepatomegaly	2
Aspiration pneumonia	1
Dyspnea	1
Chronic dermatitis and folliculitis	1
Urine and fecal incontinence	1
Pigmenturia	1

### 2.2 Muscle biopsies

**FIGURE 2:**
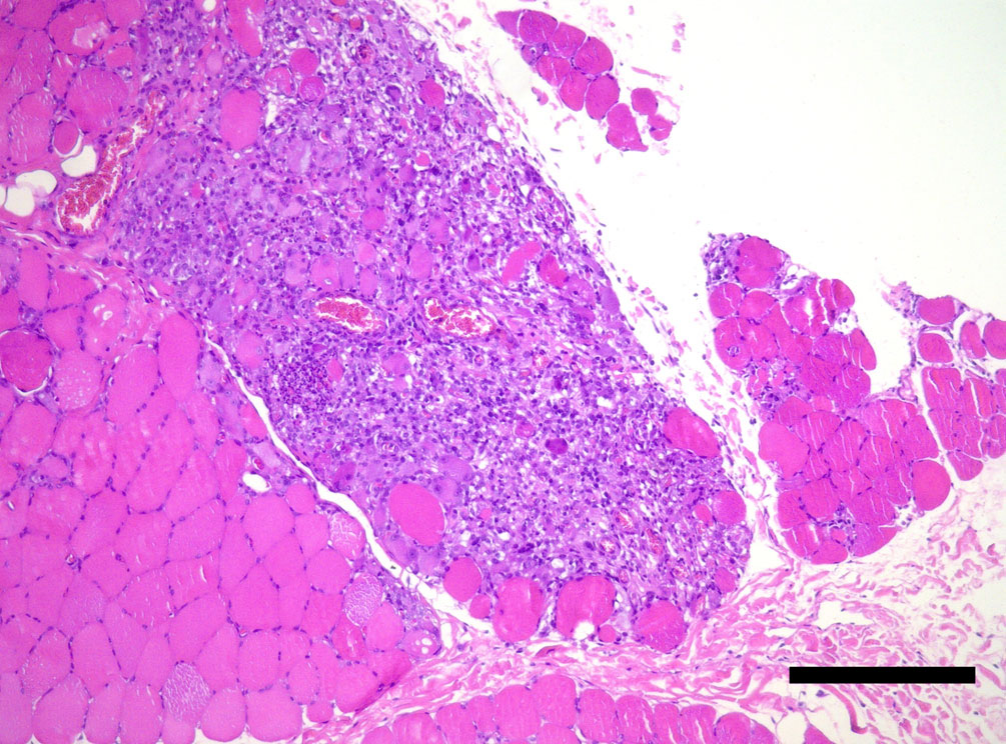
Transverse section of skeletal muscle featuring severe, diffuse, interstitial, and fiber‐directed, mixed, predominantly lymphohistiocytic infiltrates with subtotal effacement of the muscle fascicle. There is no evidence of intralesional protozoa. Case 4, paraffin section, stained with HE, ×10. Scale bar = 300 μm.

**FIGURE 3:**
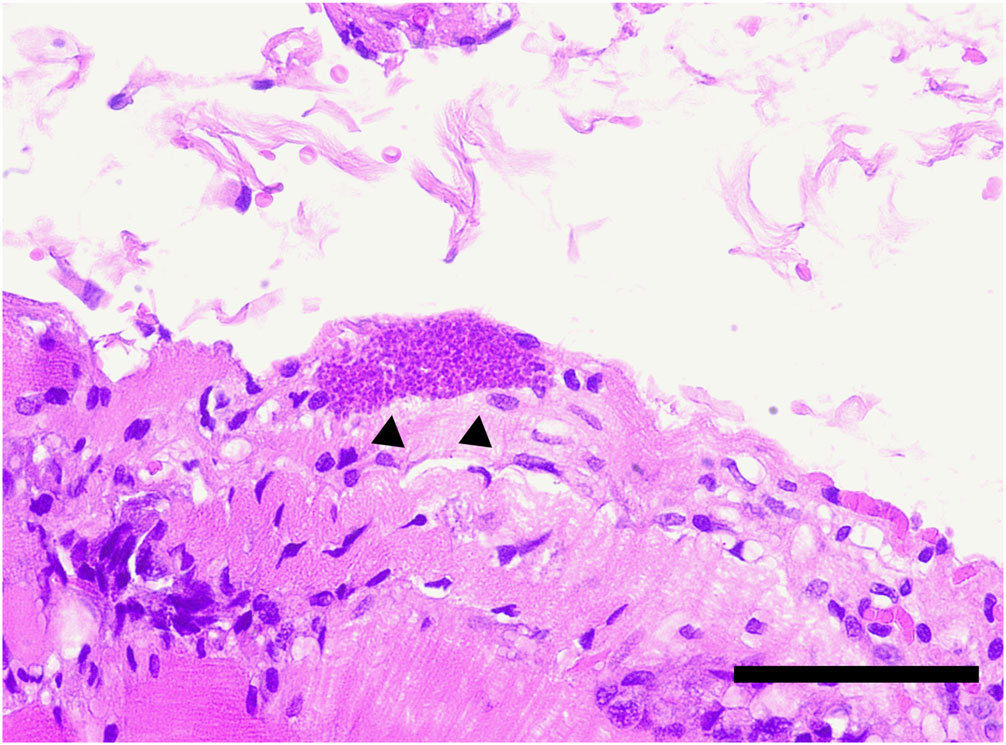
Longitudinal section of skeletal muscle from 1 infected animal. Within the sarcolemma of 1 myofiber there is a strongly basophilic parasitic cyst of several μm in length containing numerous bradyzoites (arrows). Case 8, paraffin section, stained with HE, ×40. Scale bar = 100 μm.

**FIGURE 4:**
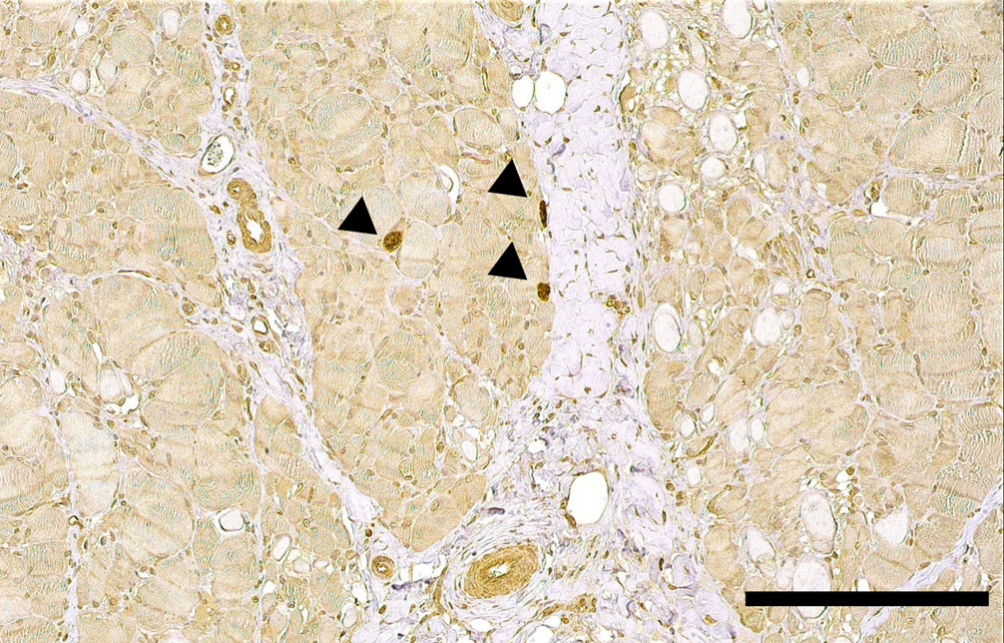
Immunohistochemistry for *Neospora caninum*. Tissue cysts containing bradyzoites can be identified through a yellow‐brown signal (arrows). Case 10, paraffin section, transverse section of skeletal muscle, stained with diaminobenzidine (DAB), ×20. Scale bar = 200 μm.

**FIGURE 5:**
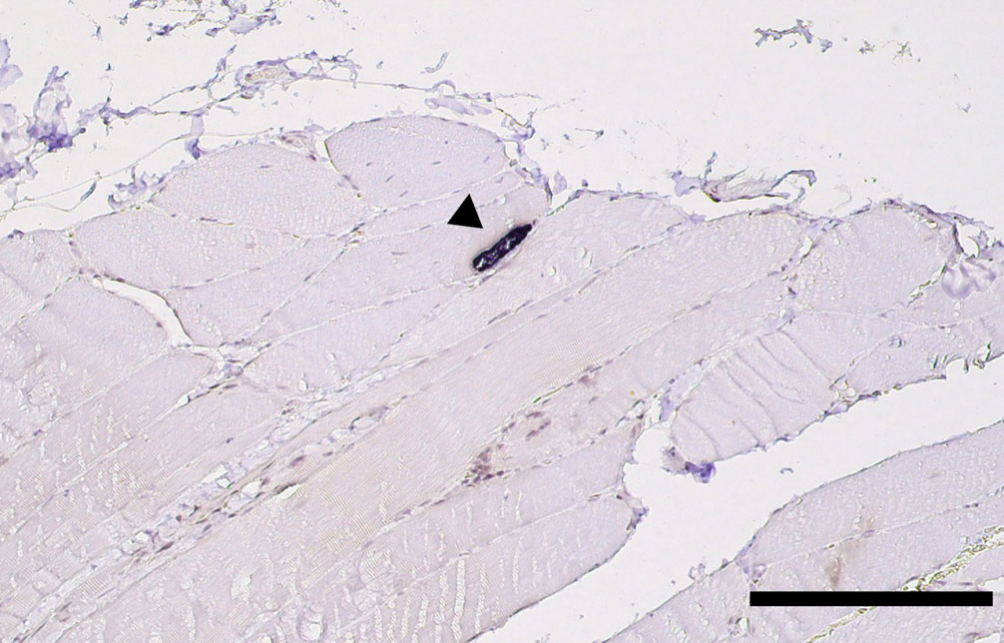
In situ hybridization for *Neospora caninum*. A single tissue cyst displays an intense dark purple signal and is easily detectable in this low magnification. Case 13, paraffin section, longitudinal section of skeletal muscle, stained with color substrates 5‐bromo‐4‐chloro‐3‐indolyl phosphate (BCIP) and 4‐nitro blue tetrazolium chloride (NBT; Roche Diagnostics), ×20. Scale bar = 200 μm.

Muscle biopsies were collected via surgical procedures performed under general anesthesia from multiple referral centers within Europe. Sent‐in tissue was snap‐frozen in isopentane and cooled in liquid nitrogen for cryohistology. Part of the samples was immersed in 10% neutral‐buffered formalin‐fixed paraffin‐embedding (FFPE). Sections were taken with a thickness of 3 to 5 μm and performed in transverse and longitudinal orientation. Cryosections were stained with HE, Engel's modified Gomori trichrome stain, oil red O, and periodic acid Schiff reaction. Nerve biopsies were fixed in 2.5% glutaraldehyde for semithin histology, on epoxy sections. Immunohistochemistry (IHC) and ISH were executed on FFPE sections. Transmission electron microscopy was performed only when considered necessary (Figures 2, 3, 4, 5).

### 2.3 Nerve biopsies

Nerve biopsies were either submitted as fresh unfixed and sent cooled overnight or prefixed in 40% formaldehyde buffered solution. Fresh samples were fixed in 2.5% glutaraldehyde for 1 hour and preserved in 0.1 M Soerensen's phosphate buffer until further processing. Regardless of fixation method, 4 segments of 2 mm length were harvested from proximal and distal edges using a razor blade. They were postfixed for 2 hours in 2% osmium tetroxide followed by repeated buffer rinses and ascending alcohol series ensued by embedding in epoxy resin. Semithin sections (0.5 μm) were mounted on standard slides and stained with Richardson staining modified by Wieczorek^28^ Osmificated intermediate parts of the fascicle, measuring ~8 mm in length, were subjected to nerve fiber teasing (NFT) with and without counterstaining by hematoxylin. Nerve fiber teasing was performed after stepwise incubation in 70% to 100% glycerol, using a dissection microscope. At least 100 osmium tetroxide stained fibers containing ≥3 internodes each were prepared for evaluation.

### 2.4 Serology and PCR

Serology was performed in different commercial laboratories depending on the referral center. For further details see Table S1 for validation and references of the diagnostic methods used in this study. A serological cutoff for IFAT positivity was set at 1 : 160 as proposed by most commercial laboratories. Dubey et al^8^ summarize that dogs with clinical *N. caninum*‐associated myositis, polyradiculoneuritis, and encephalomyelitis had IFAT titers of 1 : 50 to 1 : 200, while the suggested 1 : 800 cutoff^10^ should not be applied for the diagnosis of neuromuscular neosporosis.

The PCR was performed on FFPE muscle samples at Laboklin with a TaqMan RT‐PCR, after isolation of the material by a QIAamp DNA FFPE Tissue Kit (Qiagen).^29^ In addition, a *Toxoplasma* PCR was requested to check for *T. gondii* (co‐)infections in all cases.

### 2.5 Histopathology

All tissues and slides were investigated by at least 2 diagnosticians under light microscope (Zeiss Axioplan) according to standard diagnostic algorithms for the interpretation and reporting of neuromuscular biopsies.

### 2.6 Immunohistochemistry

IHC was performed for identifying *N. caninum* within the muscle tissue using goat anti‐*Neospora* (anti‐*N. caninum* antiserum, 1 : 400, VMRD No. 210‐70‐NC) primary antibody.

Sections were taken from paraffin blocks with a thickness of 5 μm. After deparaffinization and rehydration through xylol and a graduate alcohol series, the slides were rinsed with distilled water and boiled in a citrate‐buffer (pH = 6) for 30 minutes in the microwave for antigen retrieval. To prevent endogen peroxidase activity, the tissue was treated with 1.5% H_2_O_2_ for 10 minutes at room temperature. After rinsing in distilled water, a blocking buffer containing phosphate‐buffered saline (PBS), 1% bovine serum albumin, 0.1% TritonX 100 (Roth 3051.3), 0.2% goldfish gelatin (Sigma G7047), 0.02% NZ acid, and 2.5% normal horse serum (Vector S‐2000) with avidin (avidin/biotin blocking kit, SP‐2001, Vector Laboratories) was used. Sections were washed with PBS and incubated with the primary antibody diluted in the blocking buffer with biotin (avidin/biotin blocking kit, SP‐2001, Vector Laboratories) at room temperature for 1 hour. Next, the specimens were washed with PBS and incubated with the secondary antibody (rabbit anti‐goat immunglobulin G [IgG] antibody, biotinylated, BA‐5000‐1.5, Vector Laboratories) diluted in blocking buffer with a concentration of 1 : 400 for 30 minutes at room temperature. The sections were rinsed in PBS, treated with ABC complex (VECTASTAIN Elite ABC‐HRP Kit, PK‐6100, Vector Laboratories) for primary antibody detection, rinsed again and color reaction was realized with 3,3‐di‐amino‐benzidin (ImpactDAB Substrate Peroxidase, SK‐4105, Vector Laboratories). Then, all specimens were stained with hematoxylin, dehydrated with an alcohol series, cleared in xylol and covered.

As for quality assessment and to exclude a (co‐)infection with *T. gondii*, IHC for toxoplasmosis and positive and negative controls for each primary antibody were run in parallel.

### 2.7 In situ hybridization

Two chromogenic‐labeled probes targeting the 18S and the 28S ribosomal RNA (rRNA) of *N. caninum* were designed using homology comparisons with GenBank sequences of both *N. caninum* rRNA genes. To exclude homologies with other closely related cyst‐forming apicomplexans and to avoid cross‐reactivity, both selected probes were checked by in silico Basic Local Alignment Search Tool (BLAST) analysis.

For the selected 18S rRNA probe, there was no nucleotide mismatch to the corresponding gene sequences of *Hammondia heydorni* and *Hammondia triffittae*, but there was 1 mismatch to the 18S rRNA gene sequences of *T. gondii* and *Hammondia hammondi*. The 28S rRNA probe had 1 nucleotide mismatch to the corresponding gene sequences of *Hammondia hammondi* and *Hammondia triffittae*, 3 mismatches to *T. gondii* and 4 to *Hammondia hammondi*. The in silico analysis could not completely preclude cross‐reactivity between these protozoa.

Other closely related apicomplexan, such as *Besnoitia* spp. or *Sarcocystis* spp., had 5 or more mismatches in the corresponding gene sequences for both *N. caninum* probes, so a cross‐hybridization was not expected because of wider nucleotide differences.

*Hammonida* spp. is not reported to induce neuromuscular disease in dogs, and only an association with gastrointestinal signs (diarrhea) is known.^30^, ^31^ In contrast, *T. gondii* must be considered as differential diagnosis, so the specificity of both *N. caninum* probes was tested with *T. gondii*‐positive control tissue of squirrel monkey liver and lung.

The following oligonucleotide sequences, which are complementary to regions with complete *N. caninum* homology, were used as probe sequences:

18S RNA: 5′ CCACACAATGAAGTGTGAAGAAATCCAGAAGG 3′ and
28S rRNA: 5′ ACAAGTCAACAGCACAGTGAAGAGCAGTTG 3′

For the ISH, the protocol published by Meixner et al was applied without changes.^32^

Brain tissue of a *Neospora*‐infected dog containing numerous tissue cysts was used as a positive control. Both ISH probes were applied in combination to attain a reliable stating.

The same protocol from Meixner et al was used for the ISH control for a *T. gondii* (co‐)infection.^32^

### 2.8 Evaluation

The HE‐, IHC‐, and ISH‐stained slides were interpreted by 1 board‐certified veterinary pathologist under light microscopy (Zeiss Axioplan). In routine HE stained slides detection of inflammation, myonecrosis, related tissue damage, and the presence of tissue cysts were evaluated. The IHC and ISH for neosporosis were considered positive, when bradyzoites, tachyzoites, and/or tissue cysts could be clearly identified. The success rate of the different modalities in diagnosing neosporosis in this case series was ultimately compared and discussed. Because of the absence of a single gold standard diagnostic test, classification of dogs as being affected by neosporosis was based on the compounding evidence provided by multiple tests and clinical follow‐up. In the absence of a pre‐defined reference standard test, the Food and Drug Administration recommends expressing sensitivity as “positive percent agreement” reflecting that the estimates are not of accuracy, but of agreement of the considered test with the non‐reference standard (evidence‐based diagnosis).^33^, ^34^ Upon revision of the “Statistical Guidance on Reporting Results from Studies Evaluating Diagnostic Tests,”^34^ we expressed test performance as a percentage of positive dogs identified by each test modality.

## 3 RESULTS

### 3.1 Clinical cases

In the timeframe considered, the laboratory received 1162 muscle biopsies from dogs referred because of signs of neuromuscular disease. Myositis/necrotizing myopathy was diagnosed in 217 cases of which 19 were suspected to be caused by apicomplexan infection. Three of these latter were excluded because other etiologies could be identified as causative agents (*T. gondii*) and the remaining proved to be caused by *N. caninum*. A total of 16 dogs of various breeds and those living in different European countries (Italy [6], Germany [5], the United Kingdom [3], and Switzerland and Finland [1 each]) were identified as infected with *N. caninum*, 10 were male and 6 female. Age at presentation ranged between 2 months and 10 years (median 10.5 months, ~0.875 years). Reported clinical signs are summarized in Table 1.

The neurolocalization was considered to be generalized neuromuscular in 13/16 (81%) and combined PNS with CNS involvement in 3/16 (19%) comprising: prosencephalon, central vestibular system, and multifocal (cerebellum and brainstem). Four dogs were 7 years or older and 2/4 of them displayed PNS signs and 2/4 combined PNS and CNS involvement. All dogs below 1 year of age (9/16) presented with signs of PNS disorder.

The time between onset of clinical signs and presentation ranged from 5 days to 5 months (median 3 weeks) and this information was available for 13 of the 16 cases. The first reported differential diagnoses comprised inflammatory‐infectious because of *N. caninum* (7/15), metabolic diseases (5/15), undefined inflammatory diseases (4/15), degenerative lesions (3/15), hereditary conditions (3/15), muscular dystrophy, neoplasia, trauma, and intoxication (1/15).

### 3.2 Diagnostic workup

The results of the clinicopathologic investigation are summarized in Table S2.

CSF was examined in 8 of the 16 cases (3/8 with combined CNS and PNS neurolocalization and 5/8 with only PNS signs). Total protein concentration was increased in 4/8 (range: 369‐893 mg/L; median: 733 mg/L; ref.: <300 mg/L), 4 dogs showed mixed cellular pleocytosis, 1 had mononuclear pleocytosis and for another 1 the total nucleated cell count was inconspicuous. For 2 dogs, only the PCR results for *N. caninum* from CSF were available for review.

Electrodiagnostic data were available to varying degrees for 11 of the 16 dogs; electromyography (EMG) was performed in all 11 dogs. Electromyography identified spontaneous electric activity with positive sharp waves and fibrillation potentials in 7 cases mainly in the appendicular muscles and the spontaneous activity was detected also in the cervical paraspinal muscles in 2 of them. The muscles of the pelvic limbs were more affected than the muscles of the thoracic limbs in 4 cases and the intensity of the spontaneous activity was more severe in the distal muscles in 1 dog and equally distributed in all appendicular muscles tested in the remaining dogs. More specifically, positive sharp waves were reported in most of the front and hindlimb muscles (4/9). Fibrillation potentials were described as diffuse in 3 cases, presented as severe in the cervical paraspinal muscles of 1 dog and moderate in the remaining muscles of the other 2.

### 3.3 Magnetic resonance imaging

Magnetic resonance imaging (MRI) investigation of the brain was performed in 3 cases with combined neuromuscular and CNS signs. Case no. 3 displayed multifocal intra‐axial lesions of the brain compatible with encephalitis. Similarly, case no. 13 displayed multiple intra‐axial lesions suggestive of an infiltrative/inflammatory‐infectious etiology. Information about MRI of case no. 12 was more detailed and revealed hyperintense rim of the subdural space around the cerebellum in the T2 sequence accompanied by widening of the cerebellar folia. The changes appeared isointense to hypointense in the T1 sequence, and in the FLAIR, there was clear hyperintensity at the periphery of the cerebellum together with marked contrast enhancement of the same area compatible with an inflammatory‐infectious state. Moreover, the right masseter muscle appeared markedly atrophic, and the entire masticatory muscles showed significant contrast enhancement. The skeletal muscles of the upper cervical tract showed normal signal intensity.

### 3.4 Muscle and nerve biopsy

Muscle histology revealed expansion of the interstitium with epimysial and perimysial fibrosis in 9/16 cases (7/9 mild; 2/9 moderate) and fat replacement in 3/9. Inflammatory infiltrates were found in all cases and were defined as mild (3/16), moderate (5/16), moderate to marked (3/16), or marked (5/16), while the distribution was interstitial and fiber‐directed in all specimens. Inflammatory cell composition was lymphohistiocytic (10/16), mixed cellular (5/16), and histiocytic in 1 case. Myofiber necrosis was seen in 12/16 cases and myophagocytosis was detected in 6/16. Parasites were visualized in 9 dogs, located either in the sarcoplasm (5/9) or clustered among myofibers (4/9). Apicomplexal inclusions were found with and without accompanying inflammatory infiltrates.

Morphologic diagnosis was consistent with myositis in 10/16 (63%; 95% confidence interval [CI]: 0.39‐0.82), borderline changes in 1/16 (6%; 95% CI: 0.01‐0.28) and necrotizing myopathy in 5/16 (31%; 95% CI: 0.14‐0.56), and with reported intralesional apicomplexa in 9/16 (56%; 95% CI: 0.33‐0.77). Nerve biopsies were performed in 11/16 and revealed signs of neuropathy in 6/11 (55%; 95% CI: 0.28‐0.79). One sample was nondiagnostic. A Wallerian degeneration type of neuropathy was observed 4/11 while other samples revealed demyelinating neuropathy (1/11), unclear neuropathy (1/11), and no changes in 4/11. One sample displayed granulomatous inflammation of the peripheral nerve.

### 3.5 Immunohistochemistry

The IHC for *N. caninum* was considered positive in 9 of the 16 cases (56%; 95% CI: 0.33‐0.77) and negative in the remaining 7.

### 3.6 In situ hybridization

The ISH detected *N. caninum* in 9/16 cases (56%; 95% CI: 0.33‐0.77). In 5/16 dogs it was considered negative and in 2 cases the results of staining were not conclusive.

### 3.7 PCR

The PCR analysis from muscle samples detected *N. caninum* DNA in 11/16 (69%; 95% CI: 0.44‐0.86), in 5/16 cases PCR was negative. PCR from CSF matrix was performed in 5 cases and came back as positive in 4/5. Out of 3 cases with combined PNS and CNS involvement only 2 showed CSF PCR positivity. The liquor‐negative dog (case no. 8) displayed parasitic DNA from muscle samples.

### 3.8 Serology

Serology results were available for review in 15/16 cases and in all IFAT was performed. Although reference values for antibody tests varied depending on the laboratory, serology at time of admission was considered positive (antibody titer >1 : 160) in 10/15 and negative in 5/15, with subsequent seroconversion in 2 out of the 5 negative dogs at admission (1315; 87%; 95% CI: 0.62‐0.96; range of titers: 1 : 160‐1 : 800; median of highest titers: 1 : 320). One dog (case 16) with a negative serological result for *N. caninum* had a slightly elevated *T. gondii* IgG titer of 1 : 128.

### 3.9 Therapy and outcome

Information about therapies was available for 14 dogs. Initial treatment before biopsies comprised antibiotic therapy (3/14), prednisolone (8/14), NSAID (4/14), vitamin B (1/14), and cyclosporine (1/14). Antibiotics chosen included clindamycin in 1/3 and amoxicillin‐clavulanic in 2/3. Four dogs were hospitalized and 2 had physiotherapy. One animal required treatment with oxygen and a gastric tube for nutritional supplementation. Further information regarding treatments and their modifications in the course of the disease are reported for each case, whenever available for review, in Tables S3 and S4. Seven dogs were euthanized because of worsening of the clinical signs, relapse, or no improvement; 7 dogs recovered; 1 dog died because of the disease; and 1 animal was lost to follow‐up.

The total duration of clinical signs from onset to recovery or euthanasia/death ranged from 24 to 278 days (median: 52 days). Recovered dogs had a duration of clinical signs ranging from 60 to 278 days (median: 98 days) with 6/7 receiving antibiotic treatment, 3/7 glucocorticoids, and 1/7 cyclosporin. Two dogs were treated with clindamycin for 12 weeks, 3 for 8 weeks, and 1 for 7 weeks. Trimethoprim/sulfadizine (TSO) was administered to 2 dogs for 8 weeks and 1 for 7 weeks. Two dogs were treated with amoxicillin/clavulanic acid, but no information about the duration of administration was available. Prednisone was used in 2 dogs with no information about the duration and in 1 dog for ~9 months in combination with phenylbutazone. This dog received additional cyclosporin for 3 weeks. All cases in the recovery group had signs of PNS disorder without evidence of CNS involvement.

Of the 8 dogs that were euthanized or died, the total duration of clinical signs ranged from 24 to 192 days (median: 60) with 5/8 receiving antibiotic treatment, 6/8 glucocorticoids, and 1/8 cyclosporin. Two dogs received clindamycin for 4 weeks, and 1 for 10 days before exitus. Two other dogs had TSO for 1 and 2 months. One dog was treated with prednisone for several months, 2 dogs for 2 and 3 months, 1 for 3 weeks, and another 1 for 1 week, and 1 dog for 3 days. One animal received additional cyclosporin for 2 months. In this group with poor outcome, 3/8 had combined PNS and CNS involvement and the other 5 only had signs compatible with PNS disease.

### 3.10 Performance review of diagnostic tests

The proportion of *N. caninum*‐infected dogs correctly identified as positive was 87% with serology, 69% with PCR, and 56% with IHC, ISH, and parasite identification on standard histopathology stain (H&E). In 4 cases, *N. caninum* infection was confirmed with all 5 diagnostic methods, 3 cases with 4, 2 cases with 3, 6 dogs with 2, and 1 animal with 1 diagnostic method. All diagnostic approaches are summarized in Table S5. In the good outcome group, 5 dogs were positive in 2 tests and 1 dog each in 3 and 4. Serology was positive in all cases, ISH in 4, IHC in 3, PCR positivity and protozoa detection in histopathology in 2. Considering the group with poor prognosis, 1 animal each had 1, 2, and 3 positive tests, 2 dogs had 4, and 3 dogs had 5 positive test results. PCR detected *N. caninum* DNA in all animals, histopathology revealed protozoa in 6, serology and IHC were positive in 5, and ISH in 4.

## 4 DISCUSSION

The present study investigated retrospectively the clinical and histopathological findings of neuromuscular neosporosis in dogs and compared different diagnostic modalities in regard to analyzing diagnostic efficacy. In addition, a chromogenic ISH assay for the identification of *N. caninum* in paraffin‐embedded tissues was developed and evaluated.

The initial suspected differential diagnoses varied and were mainly focused on inflammatory/infectious and metabolic/toxic causes, reflecting a heterogeneous clinical presentation and challenges for an antemortem diagnosis.

The median age of onset was 10.5 months with several outliers up to 10 years of age. This is in contrast to the existing literature, where neuromuscular neosporosis is classically considered a disease of puppies while adult animals have asymptomatic infections.^8^, ^10^ Similar to previous reports,^2^, ^8^, ^35^ all dogs of this cohort younger than 12 months had a neuromuscular localization while, all dogs with concomitant CNS involvement mainly were older than 2 years. Occurrence in puppies is mainly related to in utero infection, while older dogs can develop neosporosis in different ways including reactivation of a latent infection by immunosuppression, a coinfection or gestation, or a combination of them.^2^ Specific factors influencing the route of infection could not be identified and information about littermates were available in 3/16. In 1 case, 1 of the siblings died because of uninvestigated reasons whereas the other 6 were healthy at time of writing. In the other 2 cases, the siblings were unaffected. Not all pups of a litter must be infected and/or manifest clinical disease, hence exclusion of *N. caninum* from the list of differential diagnoses should not be based on anamnestic data or signalment.^27^

The main clinical signs were consistent with a neuromuscular localization and comprised reduced spinal reflex activity, tetraparesis, muscle atrophy, muscle pain, and progressive paresis. Only 2 dogs had muscle contractures. However, signs of systemic illness such as hyperthermia, hepatomegaly, megaesophagus, lymphadenopathy, and enteropathy were also described in this cohort. Similar findings are also reported in the literature^2^, ^10^, ^36^, ^37^ and despite being unspecific, they suggest a wider spread of parasitic infection outside of the nervous system. It remains unclear, whether the clinical severity of neosporosis is related to the virulence of *N. caninum*, the animals' susceptibility, the immune status, or a combination of all these factors.^8^

Blood analysis provided unspecific changes indicative of systemic inflammation/infection manifested as moderately increased white blood count (WBC) in 3 dogs and increased canine C‐reactive protein in 5/9 dogs in which it was tested. On the other hand, muscle enzymes comprising CK, AST, and alanine aminotransaminase (ALT) confirmed muscle damage in 15, 12, and 9 dogs, respectively. Recently, a correlation between *N. caninum*‐induced meningoencephalitis and high CK‐ and AST activities has been demonstrated and should increase suspicion of *N. caninum* infection until other diagnostic results are available.^38^ Altogether, blood analysis can be suggestive, but cannot be reliably used as a confirmation of the infection^9^ and do not exclude degenerative processes, metabolic diseases, or trauma.

Because of the lack of effective medication and vaccines, early parasite detection is crucial for the diagnosis and for a successful outcome in canine neosporosis.^39^ Direct and indirect diagnostic modalities can confirm *N. caninum* infection in clinical practice. Titers of antibodies against *N. caninum* determined with either IFAT or ELISA reveal a high specificity, but low positive percent agreement, and might give false negative results.^40^, ^41^ IFAT titers >1 : 50 confirm exposure to the antigen and titers ≥1 : 200 are regularly seen in clinical neosporosis.^27^, ^41^, ^42^ Nevertheless, interinstitutional differences in the test procedures might impair the direct comparison of IFAT titers.^43^

In our cohort, serology was able to correctly identify 87% of dogs affected by neosporosis translating into 2/15 dogs identified as false negative. In the early stage of infection or in chronic disease, antibody‐titers can be negative. Moreover, reliable results are limited to immunocompetent animals and the detection of antibodies only confirms pathogen contact and not a disease state.^9^ Even in histologically confirmed neosporosis, low antibody titers are reported, and serologically positive dogs present with a wide range of titers, from 1 : 50 to more than 1 : 12:800.^14^, ^27^, ^44^, ^45^, ^46^ Also, no correlation has been found between antibody level and severity of the disease.^8^ Re‐evaluation of titers for seroconversion requires weeks and can result in delayed treatments and, thus, worse outcomes. Despite these caveats, serology performance (87%) of positive results on the tested cohort proved higher than that obtained with direct methods of parasite identification including PCR (69%), histopathology, IHC, and ISH (all 56%), probably because parasite content in muscle biopsies is subjected to higher variations and is influenced by sampling.

*N. caninum* DNA can be detected antemortem by PCR in several organs, blood, feces, CSF, and muscle tissue.^8^, ^47^, ^48^, ^49^ To the authors' knowledge, no studies have been published on comparing the effectiveness of *N. caninum* detection with PCR from CSF, muscle biopsies, and other tissue samples. The most accessible matrix to sample is blood, but especially in chronic disease, it is questionable if the parasite can be found in detectable amounts within the blood circulation, or more likely, as bradyzoites and tachyzoites in tissues. Depending on the clinical signs (CNS vs neuromuscular), a CSF‐tap or a muscle biopsy is advisable to collect parasitic DNA for analysis. Distribution and concentration of the parasite and its DNA on collected matrices will, however, invariably affect the accuracy of every direct identification method.

Histopathology revealed inflammatory myositis in 63%, necrotizing myopathy in 31%, borderline changes in 6%, and neuropathy in 55%. Direct visualization of bradyzoites on conventional stains (HE) occurred in 56% (9/16) of cases, including the case with only borderline changes, and in further 2 cases, an apicomplexan infection was suspected by the pathologist despite lack of identifiable parasites. Distribution of parasites among skeletal muscles, and even within a muscle sample, is not uniform and tachyzoites, bradyzoites, and tissue cysts of *N. caninum* and *T. gondii* look similar under light microscopy.^50^ Nevertheless, histopathology is a valuable method to estimate type and extent of skeletal muscle lesions, identify or at least suspect apicomplexan infection, and to exclude other causes of myopathies antemortem. Histopathology on conventional stain identified 56% of infected dogs and the same held true for IHC and ISH with 9/16 positive cases each. However, within this cohort, the positive versus negative status of the case among conventional (HE) and special (IHC; ISH) stains was not consistent and their combination allowed for identification of parasites in 13/16 dogs. Most likely, recuts of the same paraffin block disclosed parasites located at different depths within the sample, nevertheless this combined approach increased the total number of correctly diagnosed cases. To the authors' knowledge, ISH on canine muscle tissue to detect *N. caninum* has not been documented in literature so far. Apart from the lower cost of oligonucleotide probes over that of primary antibodies for IHC, ISH provides a clearer chromogenic signal from targeted structures against background tissue allowing for parasites identification even from ruptured tissue cysts, that would be otherwise challenging to achieve on HE and IHC.

The limitations of this study primarily stem from its retrospective design and the heterogeneous clinical evaluations conducted by different referral centers. As a result, there are incomplete data sets, and a standardized reference laboratory for serology and clinicopathological examinations was lacking. In addition to financial limitations, factors such as owner compliance and length of treatment plans might have influenced variables such as disease duration as well as survival. Furthermore, our study cohort was relatively small, emphasizing the importance of a larger number of cases, particularly for validating our diagnostic approach in course of suspected neuromuscular *N. caninum* infection. In conclusion, we propose a multimodal diagnostic approach to the diagnosis of *N. caninum* with neuromuscular manifestation in dogs. This approach involves a combination of at least 2 different (direct and indirect) methods starting with serology and muscle biopsy. This strategy increases the likelihood of an early and successful parasitic detection. In this cohort, diagnosis was reached with 1 test in 1/16 dogs, 2 tests in 6/16 dogs, 3 tests in 2/16, 4 tests in 3/16, and 5 tests in 4/16 dogs. When financial constraints and practicability reduce the range of choices, serology is inexpensive, easy to perform, and can provide a rapid first screen. Muscle biopsies and histopathologic investigation can confirm the presence of necrotizing myopathy/myositis with or without apicomplexan parasites by direct visualization and should provide guidance about the necessity of further tests for confirmation/exclusion. Despite the limited data available on treatments and the small cohort analyzed, the group with poor outcome appears to have received glucocorticoids more frequently, particularly before biopsy and final diagnosis. We cannot conclude if postponed initiation of specific treatment has detrimental effect on the outcome, but it seems reasonable to hypothesize that a precise and early diagnosis followed by appropriate treatment could increase the chances of a successful outcome in neuromuscular forms of neosporosis.

## CONFLICT OF INTEREST DECLARATION

Authors declare no conflict of interest.

## OFF‐LABEL ANTIMICROBIAL DECLARATION

Authors declare no off‐label use of antimicrobials.

## INSTITUTIONAL ANIMAL CARE AND USE COMMITTEE (IACUC) OR OTHER APPROVAL DECLARATION

Authors declare no IACUC or other approval was needed.

## HUMAN ETHICS APPROVAL DECLARATION

Authors declare human ethics approval was not needed for this study.

## Supporting information


**Table S1.** Validation and references for the diagnostic methods applied in this study.

**Table S2.** Results of clinicopathologic investigation.

**Table S3.** Duration of clinical signs and medication before and after muscle biopsy—cases with recovery.

**Table S4.** Duration of clinical signs and medication before and after muscle biopsy—cases with euthanasia/death.

**Table S5.** Summary and overview of patient demographics, diagnostic test results, and outcomes for each of the 16 dogs diagnosed with neosporosis.

**Table S6.** Course of antibody titer.
