# Supplementary material for: A multimodal approach to diagnosis of neuromuscular neosporosis in dogs
Source: J Vet Intern Med. 2024 Jul 17;38(5):2561–70. doi: 10.1111/jvim.17145 (PMC11423454; doi:10.1111/jvim.17145)
Supplement: Supplementary file 1 — Table S1. Validation and references for the diagnostic methods applied in this study. [file JVIM-38-2561-s004.docx]

**Supplementary material Table 1:**

**Validation and references for the diagnostic methods applied in this study.**

| **Diagnostic assay** | **Laboratory** | **Reference/Validation** |
| --- | --- | --- |
| **PCR** | Laboklin GmbH & Co.KG | Pereira GR, Vogel FS, Bohrer RC, et al. Neospora caninum DNA detection by TaqMan real-time PCR assay in experimentally infected pregnant heifers. Vet Parasitol 2014;199:129-135. |
| **IFAT** | Idexx Laboratories | Commercial IFAT testkit, validated by the supplier (according to ISO/IEC 17025:2017) |
|  | MyLav S.R.L.U. | External quality control measures; performance of IFAT with dilutions ranging from 1:80 to 1:1280 (1:80, 1:160, 1:320, 1:640, 1:1280). Titers up to 1:160 (e.g. 1:80, 1:160) should be considered as doubtful, whereas higher titers (e.g., 1:320, 1:640, 1:1280) should be interpreted as indicative of specific antibody reactivity. |
|  | Alomed-Müller GmbH & Co.KG | Commercial IFAT testkit, validated by the supplier |
|  | Private Laboratory of the Clinica Veterinaria Privata San Marco Srl | N.caninum serology was carried out by the indirect fluorescent antibody test  (IFAT) using the NC-1 stain of protozoan. |
| **IHC** | Institute for Clinical & Comparative Neuropathology  Veterinary Faculty  Ludwig Maximilians University | Internal quality assurance and validation |
| **ISH** | Institute for Clinical & Comparative Neuropathology  Veterinary Faculty  Ludwig Maximilians University | Internal quality assurance and validation |
