# Supplementary material for: A multimodal approach to diagnosis of neuromuscular neosporosis in dogs
Source: J Vet Intern Med. 2024 Jul 17;38(5):2561–70. doi: 10.1111/jvim.17145 (PMC11423454; doi:10.1111/jvim.17145)
Supplement: Supplementary file 2 — Table S2. Results of clinicopathologic investigation. [file JVIM-38-2561-s001.docx]

**Supplementary materials Table 2: Results of clinicopathologic investigation.**

| **Case** | **HCT (%)** | **WBC (10^9/l)** | **CRP (mg/l)** | **CK (U/l)** | **GGT (U/l)** | **ALT (U/l)** | **AST (U/l)** | **ALP (U/l)** |
| --- | --- | --- | --- | --- | --- | --- | --- | --- |
| **#1** | 38.6 | 7.99 | / | 18687 | 18 | 1543 | 1037 | 290 |
| **#2** | 45.3 | 10.0 | 0 | 1761 | / | 545 | 199 | 48 |
| **#3** | / | / | / | * | / | / | / | / |
| **#4** | 45.9 | 17.9 | 62.6 | 11651 | 4 | 5460 | 1210 | 58 |
| **#5** | / | / | / | 4368 | / | / | / | / |
| **#6** | 56.0 | 7.39 | 113.7 | 29906 | 3 | 310 | 1674 | 52 |
| **#7** | 47.0 | 20.05 | 71.8 | 309 | 11 | 58 | 3260 | 222 |
| **#8** | 30.5 | 8.01 | / | 4000 | 1 | 1000 | / | 145 |
| **#9** | 58.0 | 6.83 | / | 14511 | 2 | 791 | 2205 | 61 |
| **#10** | 32.9 | 11.15 | / | 809 | 3 | 53 | 40 | 244 |
| **#11** | 44.2 | 14.61 | / | 808 | / | 105 | 66 | 45 |
| **#12** | 44.4 | 8.95 | 16.7 | 14062 | 0 | 854 | / | 153 |
| **#13** | 40.7 | 13.23 | 0 | 6939 | 3 | 293 | 311 | 26 |
| **#14** | 35.3 | 9.48 | 1.0 | 257 | 0 | 27 | 18 | 152 |
| **#15** | 35.0 | 17.67 | 11.6 | 4221 | 9 | 498 | / | 408 |
| **#16** | 60.1 | 9.4 | 5.6 | 3325 | 4 | 445 | 243 | 34 |
| **Range, mean and median** | 32.9 – 60.1  (43.9; 44.3) | 6.83 – 20.05 (11.6; 9.7) | 0 – 113.7 (31.4; 11.6) | 257 –  29 906 (7707.6; 4221) | 0 – 18  (4.8; 3.0) | 27 – 1543 (855.8; 471.5) | 18 – 3260 (933; 311) | 26 – 408 (138.4; 103) |
| **Reference interval** | 37.3 - 61.7 | 5.05 - 16.76 | 0 - 10.0 | 0 – 463 | 0 - 5 | 8 – 75 | 0 – 50 | 46 – 337 |

**/ No data**

*** Missing data**
