# Supplementary material for: A multimodal approach to diagnosis of neuromuscular neosporosis in dogs
Source: J Vet Intern Med. 2024 Jul 17;38(5):2561–70. doi: 10.1111/jvim.17145 (PMC11423454; doi:10.1111/jvim.17145)
Supplement: Supplementary file 3 — Table S3. Duration of clinical signs and medication before and after muscle biopsy—cases with recovery. [file JVIM-38-2561-s006.docx]

**Supplementary material table 3:**

**Duration of clinical signs and medication before and after muscle biopsy – cases with recovery**

| **Case** | **Duration of clinical signs before biopsy (days)** | **Medication before biopsy** | **Duration of medication after biopsy (days)** | **Medication after biopsy** | **Duration of clinical signs and medication in total** | **Outcome** |
| --- | --- | --- | --- | --- | --- | --- |
| **#2** | 37 | Amoxicillin/Clavulanicacid | 60 | Clindamycin 11.5mg/kg TID for 8 weeks | 97 | Recovery |
| **#4** | 5 | Amoxicillin/Clavulanicacid,  Glucocorticoids |  |  |  | Recovery |
| **#6** | 180 | NSAID, Prednisolone | 98 | Clindamycin 15mg/kg BID for 8 weeks,  TSO 15mg/kg BID for 8 weeks,  Meloxicam 0.1mg/kg SID, | 278 | Recovery |
| **#7** | 10 |  | 84 | Clindamycin 11 mg/kg BID for 12 weeks, Maropitant 1 mg/kg SID,  Omeprazole 1 mg/kg BID,  vitamin supplementation | 94 | Recovery |
| **#11** | 120 | Vitamin B | 60 | TSO 400/80 (3/15 mg/kg) BID for 8 weeks,  Clindamycin 300mg (12mg/kg) BID for 8 weeks;  L-Carnitine 2.5ml TID,  Vitamin-B Complex,  Metamizole | 180 | Recovery |
| **#14** | 14 | Meloxicam | 84 | Clindamycin 20mg/kg BID for 12 weeks;  Pregabalin 25mg/kg TID,  Robenacoxib 10mg SID | 98 | Recovery |
| **#15** | 30 | Phenpred  (6mg/kg Phenylbutazon + 0,2mg/kg Prednisolone) for 8 months  Concomitant: Phenobarbital 1mg/kg BID (primary epilepsy for 4 years) | 30 | TSO 80 + 400mg (2.6/13.3 mg/kg) BID; Clindamycin 300mg (10mg/kg) TID;  Prednisolone 1.2mg/kg BID;  Gabapentin;  **2 weeks later:** considerable worsening and supposed IMPM,  stop with TSO and Clindamycin, start with additional Cyclosporin;  **3 weeks later:** further worsening, restart with TSO and Clindamycin, stop with Cyclosporin and reduction of Prednisolone;  **2 weeks later:** end of Prednisolone, slow improvement;  **2 weeks later**: clear improvement, regressive muscle atrophy; 1 week after stop with antibiotics: almost complete recovery | 60 | Recovery |
|  |  |  |  |  |  |  |
| **Range**  **(days)** | 5 - 180 |  | 30 - 98 |  | 60 - 278 |  |
| **Mean**  **(days)** | 56 |  | 69 |  | 129 |  |
| **Median**  **(days)** | 30 |  | 72 |  | 98 |  |

TSO: Trimethoprim/Sulfadizine

SID: semel in die; once a day

BID: bis in die; twice a day

TID: ter in die; three times a day
