# Supplementary material for: A multimodal approach to diagnosis of neuromuscular neosporosis in dogs
Source: J Vet Intern Med. 2024 Jul 17;38(5):2561–70. doi: 10.1111/jvim.17145 (PMC11423454; doi:10.1111/jvim.17145)
Supplement: Supplementary file 4 — Table S4. Duration of clinical signs and medication before and after muscle biopsy—cases with euthanasia/death. [file JVIM-38-2561-s002.docx]

**Supplementary material table 4:**

**Duration of clinical signs and medication before and after muscle biopsy – cases with euthanasia/death**

| **Case** | **Duration of clinical signs before biopsy (days)** | **Medication before biopsy** | **Duration of medication after biopsy (days)** | **Medication after biopsy** | **Duration of clinical signs and medication in total (days)** | **Outcome** |
| --- | --- | --- | --- | --- | --- | --- |
| **#3** | 30 | Prednisolone, Cyclosporine | 30 | Prednisolone, Cyclosporine | 60 | Euthanasia |
| **#5** | 60 | Clindamycin 150mg (11.23mg/kg) BID for 7 days,  Prednisolone 25mg (1.25mg/kg) SID for 7 days | 30 | Tapering off prednisolone,  continue Clindamycin for further 4 weeks | 90 | Euthanasia |
| **#8** |  |  |  |  |  | Euthanasia |
| **#9** | 14 | Prednisolone | 10 | Prednisolone 0.5mg/kg SID,  Clindamycin 10mg/kg BID | 24 | Death |
| **#10** | 30 |  | 30 | Clindamycin 15mg/kg BID | 60 | Euthanasia |
| **#12** | 21 | Prednisolone 5mg SID | 60 | TSO 15mg/kg BID;  Prednisolone 2.5mg/kg SID;  **Two days later**:  start with pyrimethamine 25mg (1mg/kg) SID,  reduction of Prednisolone 1.25mg/kg SID,  Vitamin B – Complex,  Metamizole | 81 | Euthanasia |
| **#13** | 180 | Prednisolone | 12 | Prednisolone | 192 | Euthanasia |
| **#16** | 14 | Meloxicam | 30 | L-Carnitin 50 mg/kg BID;  Famotidin 0.6 g/kg BID,  Prednisolone 1.6 mg/kg SID,  TSO 16mg/kg BID;  **3 days later**: stop with prednisolone, start with pyrimethamine 25mg (1mg/kg) SID | 44 | Euthanasia |
|  |  |  |  |  |  |  |
| **Range**  **(days)** | 14 - 180 |  | 12 - 60 |  | 24 - 192 |  |
| **Mean**  **(days)** | 49 |  | 28.8 |  | 69 |  |
| **Median**  **(days)** | 30 |  | 30 |  | 60 |  |

TSO: Trimethoprim/Sulfadizine

SID: semel in die; once a day

BID: bis in die; twice a day

TID: ter in die; three times a day
