# Supplementary material for: A multimodal approach to diagnosis of neuromuscular neosporosis in dogs
Source: J Vet Intern Med. 2024 Jul 17;38(5):2561–70. doi: 10.1111/jvim.17145 (PMC11423454; doi:10.1111/jvim.17145)
Supplement: Supplementary file 5 — Table S5. Summary and overview of patient demographics, diagnostic test results, and outcomes for each of the 16 dogs diagnosed with neosporosis. [file JVIM-38-2561-s005.docx]

**Supplementary material table 5: Summary and overview of patient demographics, diagnostic test results, and outcomes for each of the 16 dogs diagnosed with Neosporosis.**

| **Case** | **Age** | **Gender** | **Breed** | **Neuro-localization** | **Histologic findings** | **Parasites on light Microscopy** | **PCR** | **Serology** | **IHC** | **ISH** | **Outcome** |
| --- | --- | --- | --- | --- | --- | --- | --- | --- | --- | --- | --- |
| **#1** | 7m | f | Mix | PNS | IM | + | + | + | + | + | / |
| **#2** | 9m | f | Labrador | PNS | IM | + | - | + | + | - | Recovery |
| **#3** | 8y | f | English Springer Spaniel | Combined CNS & PNS | IM | + | + | / | - | - | Euthanasia |
| **#4** | 8m | m | Rhodesian Ridgeback | PNS | IM | + | + | + | - | + | Recovery |
| **#5** | 10m | m | Greyhound | PNS | IM | + | + | + | + | + | Euthanasia |
| **#6** | 6y | m | Fox Terrier | PNS | NM | - | - | + | - | + | Recovery |
| **#7** | 6m | m | American Staffordshire Terrier | PNS | NM | - | + | + | - | * | Recovery |
| **#8** | 3m | m | Boxer | PNS | IM | + | + | - | + | + | Euthanasia |
| **#9** | 7y 10m | m | Greyhound | PNS | IM | + | + | + | + | + | Died |
| **#10** | 2m | m | Czech Wolf | PNS | IM | + | + | + | + | + | Euthanasia |
| **#11** | 11m | f | Labrador | PNS | IM | - | - | + | - | + | Recovery |
| **#12** | 2y | f | Crossbreed | Combined CNS & PNS | BC | + | + | + | - | - | Euthanasia |
| **#13** | 7y | m | Cane Corso | Combined CNS & PNS | NM | - | + | + | + | + | Euthanasia |
| **#14** | 3m | f | Labrador | PNS | IM | - | - | + | + | * | Recovery |
| **#15** | 10y | m | Labrador | PNS | NM | - | - | + | + | - | Recovery |
| **#16** | 6.5 y | m | Saluki | PNS | NM | - | + | - | - | - | Euthanasia |
|  |  |  |  |  |  |  |  |  |  |  |  |
| **Percentage of positively tested dogs:** |  |  |  |  |  | 56.3% | 68.6% | 86.7% | 56.3% | 56.3% |  |

**+ Positive**

**- Negative**

**/ No data/not performed**

*** Unclear signal**

**IM: Inflammatory myopathy**

**NM: Necrotizing myopathy**

**BC: Borderline changes**
