# Supplementary material for: A multimodal approach to diagnosis of neuromuscular neosporosis in dogs
Source: J Vet Intern Med. 2024 Jul 17;38(5):2561–70. doi: 10.1111/jvim.17145 (PMC11423454; doi:10.1111/jvim.17145)
Supplement: Supplementary file 6 — Table S6. Course of antibody titer. [file JVIM-38-2561-s003.docx]

**Supplementary material: Course of antibody titre**

|  | **Case number** | | | | | | | | | | | | | | | |
| --- | --- | --- | --- | --- | --- | --- | --- | --- | --- | --- | --- | --- | --- | --- | --- | --- |
|  | **#1** | **#2** | **#3** | **#4** | **#5** | **#6** | **#7** | **#8** | **#9** | **#10** | **#11** | **#12** | **#13** | **#14** | **#15** | **#16** |
| **0 days** | 1:400 | 1:320 | not performed | 1:320 | 1:800 | 1:50 | 1:320 | 1:50 | 1:320 | 1:80 | 1:320 | 1:320 | 1:640 | 1:320 | 1:160 | negative |
| **2 days** |  |  |  |  |  |  |  |  |  |  |  | negative |  |  |  |  |
| **1 week** |  | negative |  |  |  |  |  |  |  |  |  |  |  |  |  |  |
| **2 weeks** |  |  |  |  |  |  | 1:160 |  |  |  |  | 1:320 |  |  | 1:400 |  |
| **3 weeks** |  |  |  |  |  |  |  |  |  | 1:160 |  |  |  |  |  |  |
| **4 weeks** |  |  |  |  |  | 1:200 |  |  |  |  |  |  |  | 1:100 | 1:320 |  |
| **7 weeks** |  |  |  |  |  |  |  |  |  |  |  |  |  |  | 1:160 |  |
| **9 weeks** |  |  |  |  |  |  |  |  |  |  |  |  |  | 1:50 |  |  |
| **10 weeks** |  |  |  |  |  |  |  |  |  |  |  |  |  | 1:160 |  |  |
| **11 weeks** |  |  |  |  |  |  |  |  |  |  |  | 1:320 |  |  | 1:80 |  |
| **4 months** |  | negative |  |  |  |  |  |  |  |  |  |  |  |  |  |  |
| **9 months** |  | negative |  |  |  |  |  |  |  |  |  |  |  |  |  |  |
| **22 months** |  | negative |  |  |  |  |  |  |  |  |  |  |  |  |  |  |
